# Aligning Video Models with Human Social Judgments via Behavior-Guided Fine-Tuning

**Published:** 2025-10-01

**Authors:** Kathy Garcia, Leyla Isik

**Affiliations:** 1 Department of Cognitive Science, Johns Hopkins University; 2 Department of Biomedical Engineering, Johns Hopkins University

## Abstract

Humans intuitively perceive complex social signals in visual scenes, yet it remains unclear whether state-of-the-art AI models encode the same similarity structure. We study (Q1) whether modern video and language models capture human-perceived similarity in social videos, and (Q2) how to instill this structure into models using human behavioral data. To address this, we introduce a new benchmark of over 49,000 odd-one-out similarity judgments on 250 three-second video clips of social interactions, and discover a modality gap: despite the task being visual, caption-based language embeddings align better with human similarity than any pretrained video model. We close this gap by fine-tuning a TimeSformer video model on these human judgments with our novel hybrid triplet–RSA objective using low-rank adaptation (LoRA), aligning pairwise distances to human similarity. This fine-tuning protocol yields significantly improved alignment with human perceptions on held-out videos in terms of both explained variance and odd-one-out triplet accuracy. Variance partitioning shows that the fine-tuned video model increases shared variance with language embeddings and explains additional unique variance not captured by the language model. Finally, we test transfer via linear probes and find that human-similarity fine-tuning strengthens the encoding of social-affective attributes (intimacy, valence, dominance, communication) relative to the pretrained baseline. Overall, our findings highlight a gap in pretrained video models’ social recognition and demonstrate that behavior-guided fine-tuning shapes video representations toward human social perception.

## 1 Introduction

Humans effortlessly perceive the visual social world with remarkable nuance: we readily distinguish whether two people are comforting each other, collaborating, or competing—all by watching brief interactions. Humans can rapidly extract abstract information about intention, affect, and context, far beyond surface-level motion or pose information (Canessa et al., 2012; Lee Masson & Isik, 2021; McMahon et al., 2023). As AI systems increasingly interpret and interact in human-centered environments, aligning their representations with human social perception is essential. Yet, it remains unclear whether state-of-the-art models perceive social similarity the way humans do.

In this work, we investigate: **(Q1)** To what extent do current pretrained video and language models capture human-perceived similarity between social videos? **(Q2)** How can we instill a more human-like similarity structure into a video model using human behavioral data?

To address these, we introduce a new dataset of 49,484 human odd-one-out (OOO) triplet similarity judgments over 250 short (3s) videos depicting everyday social scenes. Each triplet judgment identifies which of three videos is least like the others, inducing a behavioral similarity structure over the video set. Remarkably, we find that embeddings from a language model applied to video captions outperform all pretrained video model embeddings at predicting these judgments, despite the human task being presented in a purely visual manner. To close this gap, we then propose a behavior-guided fine-tuning strategy that incorporates human similarity judgments directly into video model training. We introduce a hybrid loss combining: (i) Triplet loss, enforcing local alignment with human triplet OOO comparisons; (ii) representational similarity analysis (RSA) loss, aligning the global pairwise embedding structure with human representational similarity matrices (RSMs). Using parameter-efficient low rank adaptation (LoRA) (Hu et al., 2022), we fine-tune a TimeSformer video model with *<* 2 parameter updates. Our approach substantially improves human-model alignment: fine-tuning explained variance increases by 58% relative to the pretrained baseline, approaching the behavioral reliability ceiling, and surpasses language model performance. Variance partitioning shows that the fine-tuned video model more strongly overlaps with the language model, compared to the pre-trained baseline, and explains additional variance in human judgments not captured by the language model.

### Contributions.

In this work, we make three main contributions: (1) We introduce a benchmark of ∼49k human odd-one-out judgments on social video clips, providing the first large-scale dataset of human-perceived similarity in videos. (2) We propose a geometry-level training method that combines triplet supervision with a differentiable RSA objective to directly shape video representation spaces. (3) We provide empirical evidence that behavior-guided fine-tuning achieves near-ceiling alignment with human similarity judgments, surpassing the best language model.

## 2 Related Work

### Human Similarity Judgments in Vision.

Measuring how humans perceive similarity among stimuli has long been a tool to probe mental representations (Biederman, 1987; Edelman, 1998; Nosofsky, 1986; Goldstone, 1994; Hebart et al., 2020). Large-scale behavioral studies have mapped out the “similarity space” humans use for objects and scenes. Prior work has used odd-one-out (OOO) and triplet tasks to reveal the latent structure of human perception in domains such as objects (Hebart et al., 2020), ”reachspaces” (reachable interaction environments; Josephs et al., 2023), and materials (Schmidt et al., 2025). The majority of prior work focuses on the similarity structure of static image content. Our work extends this approach to social video—an underexplored but critical domain in human vision.

One prior study has investigated human judgments of dynamic stimuli and found that these judgments rely more on social-affective features than surface visual or scene features (Dima et al., 2022). While this prior work has modeled dynamic similarity judgments it has focused on explaining human judgments rather than model alignment.

### Aligning Models with Human Perception.

There is growing interest in aligning model representations with human cognitive representations, with the goal of improving interpretability and performance. Most efforts at human-alignment rely on direct human feedback, for example reinforcement learning from human feedback for generative video or text-to-video models (Kaufmann et al., 2023; Liu et al., 2025). Such supervision optimizes task rewards or output quality, but does not necessarily constrain the internal geometry of representations. These approaches are often data-intensive/require a human in the loop (Furuta et al., 2024; Li et al., 2024).

Odd-one-out similarity judgments, in contrast, provide richer relational supervision: each triplet encodes a relative comparison that reflects latent social structure, rather than scalar preferences alone. Muttenthaler et al. (2023) show that globally aligning model similarity to human judgments yields more interpretable features, but focus on static images. Further, a recent model DreamSim (Fu et al., 2023) learns perceptual similarity from synthetic image pairs. Tuning an embedding space to these judgments produced a metric that aligned better with human perception and improved image retrieval performance. Unlike these, our work targets dynamic, naturalistic social video and injects similarity structure directly through fine-tuning. These methods underscore the value of human data, but they focus on static images, low-level perceptual features, or synthetic domains. By contrast, our work tackles *dynamic, naturalistic social videos* and injects similarity structure directly through fine-tuning.

### Beyond Categorical Video Pretraining.

Prior work has focused on large-scale pretraining and transformer-based architectures such as TimeSformer (Bertasius et al., 2021), ViViT (Arnab et al., 2021), and VideoMAE (Tong et al., 2022), which achieve state-of-the-art results on action classification benchmarks. While powerful, their training objectives emphasize categorical recognition (e.g., “dancing” vs. “cooking”) rather than higher-level aspects of social behavior such as intentions, affect, or interaction dynamics. More recent multimodal video-language models, such as VideoCLIP (Xu et al., 2021) and All-in-One (Wang et al., 2022), enrich video embeddings through textual supervision, providing access to semantic abstractions not easily derived from raw video. However, these approaches still depend on language annotations and may not directly reflect the relational or affective cues that guide human perception of social similarity. Self-supervised alternatives, such as V-JEPA (Assran et al., 2025), move beyond categorical or caption-based supervision by training predictive representations of future video content, showing progress toward capturing higher-level temporal and semantic structure. Other directions have scaled video-language alignment with large paired datasets (Rizve et al., 2024), improved robustness with contrastive caption perturbations (Bansal et al., 2023), or incorporated human preference annotations to guide generative models (Wang et al., 2024). Yet no prior work has aligned video models on the human similarity structure of dynamic social scenes.

## 3 Methods

**Figure 1:**
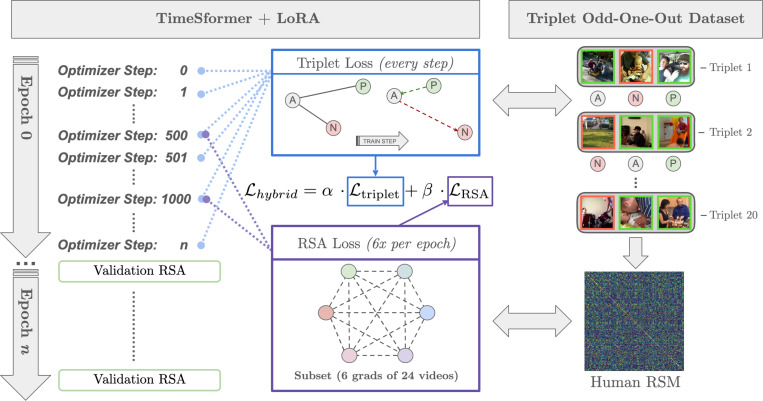
Triplet Odd-One-Out Dataset and TimeSformer Hybrid fine-tuning. We collect similarity judgments via a triplet odd-one-out task. Human choices are used as positive and negative signals for each training loss. At every optimizer step, the model is updated with a triplet loss (blue) on a batch of Anchor (A), Positive (P), Negative (N) triplets. Periodically (≈ 6 times per epoch), an additional RSA loss (purple) is applied on a small subset of 24 videos with 6 as gradients by aligning the model’s pairwise distances with the human similarity derived from all triplets. The combined training objective of triplet and RSA loss is defined in Eq. 5.

Our approach has two stages: (1) Measure human-perceived similarity – we collect odd-one-out judgments on video triplets to construct a human similarity matrix; (2) Behavior-guided fine-tuning – we fine-tune a video model so that its embedding distances better match this human similarity structure. We achieve this through a hybrid loss that enforces local triplet constraints and global alignment of similarity matrices (Fig. 1).

### 3.1 Human Similarity Judgment Dataset

We introduce a novel, large-scale dataset of human similarity judgments of short video clips. The stimulus set consists of 250 short video clips (3 seconds each) depicting a wide range of everyday human activities and social situations from a publicly available dataset (McMahon et al., 2023; Garcia et al., 2025), a subset of the Moments in Time dataset (Monfort et al., 2019), densely labeled with human social judgments. Each video was paired with a brief descriptive caption (one sentence summarizing the action) to evaluate language models.

We use a triplet odd-one-out paradigm to gather similarity judgments (Hebart et al., 2020). In each trial, a participant saw three videos (see Appendix A), and were asked to “focus on what the people are doing and choose the odd-one-out”. By choosing the odd-one-out, the participant implicitly indicated that the other two were more similar to each other. This triplet-based method yields more information per trial than a simple pairwise rating. 245 human participants were recruited online via the Meadows Research platform (https://meadows-research.com) and participated in the study. All participants gave informed consent in accordance with our internal Institutional Review Board, who provided explicit approval of all protocols and procedures discussed.

For model training and evaluation, judgments were split based on the pre-determined stimulus split released with the benchmark: 200 train videos (24,096 triplets) and 50 test videos (368 triplets). For both train and test set of judgments, we calculated a 200 × 200 human similarity matrix $S^{\left(human\right)}$ and a 50 × 50 human similarity matrix, respectively. Following prior work (Hebart et al., 2020), we define the human similarity between two videos as the probability (or frequency) that they were judged together (not odd-one-out) in triplet trials.

### Choice of Distance Metric.

Because embeddings from different architectures vary widely in scale and norm, we use cosine similarity as the primary pairwise metric. For a video v with embedding f(v), the similarity between videos i and j is:

(1) $$S_{ij}^{\text{(model)}}=\cos\left(f\left(v_{i}\right),f\left(v_{j}\right)\right).$$

Cosine similarity emphasizes the angular relationship between vectors, effectively normalizing differences in magnitude across features. This is particularly useful when comparing across layers or across different architectures, where feature norms may differ systematically. Empirically, we found that cosine similarity correlates more strongly with human judgments than Euclidean distance, in line with prior work on representational alignment (Hebart et al., 2020; Kriegeskorte et al., 2008).

### 3.2 Representations from Video and Language Models

We evaluate pretrained models on how well their layer-wise embeddings aligned with the human similarity structure (Q1). For each model layer, we obtained a feature embedding for each video (or sentence caption) and computed an analogous 50×50 similarity (or distance) matrix, for comparison to the human test set RSM with RSA (Kriegeskorte et al., 2008).

We evaluate 8 pretrained vision models including both CNN-based and Transformer-based video encoders. For example, X3D-M – a CNN from the X3D family optimized for efficient video classification (Feichtenhofer, 2020), SlowFast – a two-pathway CNN capturing both slow and fast temporal dynamics (Feichtenhofer et al., 2018); and TimeSformer – a video Transformer that factorizes spatial and temporal attention trained on Kinetics-400 (Kay et al., 2017; Bertasius et al., 2021). We feed each 3s clip into these models (after resizing frames to the required model resolution). We take the model’s embeddings at every layer, utilizing the DeepJuice software package (Conwell et al., 2024) for efficient layer-wise calculations. For fairness, we ensure each embedding is a vector of comparable dimension by down-sampling using sparse random projection (SRP) based on the Johnson–Lindenstrauss (JL) lemma with ε=0.1. This automatically sets the projection size according to the number of samples, yielding 4,732 dimensions for the training split (N=200) and 3,353 dimensions for the test split (N=50), which preserves pairwise distances within ±10% with high probability. To select the evaluation layer, we perform a 5-fold cross-validation on the 200-video training set, choose the layer with the highest mean Spearman’s ρ across folds, and then fix that layer for evaluation on the held-out 50-video test set.

For each video, we also obtain a representation from a language model based on the video’s caption. We selected 22 widely used transformer-based language models spanning sentence vs. retrieval objectives, parameter scales, and multilingual coverage, yielding a diverse and reproducible set of off-the-shelf caption encoders for comparison. (see Appendix Tab. 2) and similarly compute a similarity matrix for the captions based on cosine similarity between the layer-wise embeddings. We include the top language model’s (paraphrase-multilingual-mpnet-base-v2) alignment performance as a point of comparison to video models (Appendix Tab. 2).

### 3.3 Behavior-Guided Fine-Tuning of the Video Model

Our core approach for (Q2) is to fine-tune a video model using the human judgments as supervision. We focus on the transformer architecture with the highest pretrained performance (TimeSformer). We apply a lightweight fine-tuning strategy with LORA, updating less than 2% of the model’s parameters (1.9M trainable vs. 123M total) while keeping the other 121M parameters frozen. This approach inserts low-rank matrices into each attention layer (rank = 16), enabling efficient adaptation with minimal compute overhead and reduced risk of overfitting to our dataset.

#### 3.3.1 Hybrid Loss Function

We design a loss $L_{\text{hybrid}}$ that combines a triplet loss term $\left(L_{\text{triplet}}\right)$ and an RSA loss term $\left(L_{\text{RSA}}\right)$ to address both local and global alignment (Fig. 1).

##### Shared notation and distance.

Let f(v) be the embedding of video v. We use $\ell_{2}$-normalized embeddings $z_{i}=f\left(v_{i}\right)/{\left\|f\left(v_{i}\right)\right\|}_{2}$ and define a single cosine-distance operator that is shared by both losses:

(2) $$d(i,j)=1-\langle z_{i},z_{j}\rangle .$$

##### Triplet Loss (local constraints)

For each human odd-one-out judgment we seek to minimize the distance between anchor video i and its positive pair j to be less than the distance to its negative pair k (odd-one-out) by a margin of γ. Specifically, we penalize violations of a margin γ=0.2:

(3) $$L_{\text{triplet }}(i,j,k)=\max\{0,\;d(i,j)-d(i,k)+\gamma \}.$$

##### RSA Loss (global geometry)

To shape the broader geometry toward human similarity structure, we inject an RSA step six times per epoch. At each RSA step, we sample a batch of K=24 videos K and designate a subset of M=6 indices G⊂K whose embeddings carry gradients. We limit gradients to M=6 to keep memory and runtime manageable while still providing ample supervision: each RSA step considers all pairs that include one of these six videos (up to 123 pairs before masking), which we found gives a strong signal without the overhead of updating all 24 items.

We calculate model RDM entries with d(⋅,⋅) for all unordered pairs {i,j}⊂K with i≠j and i∈G or j∈G. Corresponding human distances $d^{\text{H}}(i,j)$ are taken from the split-specific behavior RDM, masking out pairs without judgments to create a masked index set M.

The RSA loss is the negative RSA score between the z-scored model and human distances of the masked index set M:

(4) $$L_{\text{RSA}}=-\mathrm{corr}\left(z(\mathrm{vec}(d))[M],z\left(\mathrm{vec}\left(d^{\text{H}}\right)\right)[M]\right),$$

where vec(⋅) denotes vectorization of the upper triangle, and z(⋅) denotes per-step standardization to zero mfean and unit variance.

Pearson correlation is used for the RSA loss during training to ensure the loss is differentiable.

##### Hybrid Loss.

We combine the triplet (local) and RSA (more global) supervision with a weighted objective:

(5) $$L_{\text{hybrid }}^{\left(t\right)}=\alpha {L_{\text{triplet }}}^{\left(t\right)}+{⊮}_{\text{RSA}}(t)\beta {L_{\text{RSA}}}^{\left(t\right)},$$

where $L_{\text{triplet }}$ captures fine-grained constraints from odd-one-out judgments and $L_{\text{RSA}}$ encourages broader geometric alignment on sampled subsets. The indicator ${⊮}_{\text{RSA}}(t)$ equals 1 if step t is one of the scheduled RSA steps and 0 otherwise. Specifically, we compute the total number of optimizer steps in an epoch, divide by 6, and activate the RSA loss at these evenly spaced intervals. We fix α=0.7 and linearly ramp β from 0.3 to 0.7 over training epochs.

##### Training Procedure.

We fine-tune for 50 epochs with AdamW (see Loshchilov & Hutter, 2017) with learning rate = 1×10^−4^, mixed precision, and gradient-checkpointing, using a batch size of 4. At each optimizer step, we apply the triplet loss; the RSA term is injected periodically as described above. We select the best checkpoint by RSA validation performance on a held-out 20% split of the training judgments (monitoring explained variance $R^{2}$). For ablations, we also train models with triplet-only and RSA-only objectives under the same optimizer and schedule.

#### 3.3.2 Out-of-distribution Linear Probes for Social-Affective Attributes

To see if human similarity alignment improves the model’s human alignment with other, out-of-distribution, tasks, we use human annotations for five key attributes of social scenarios included in the video dataset (McMahon et al., 2023): *Intimacy* (how intimate/personal the interaction is), *Valence* (overall emotional positivity vs negativity), *Arousal* (energy or intensity of the action), *Dominance* (power dynamic between people), and *Communication* (whether people in the video are communicating with one another). Multiple annotators independently rated, averaged, and *z-*scored each of the 250 videos on these scales. We use a ridge regression linear probe on layer-wise model embeddings with the same train-test split for the models as main experiments.

#### 3.3.3 Action-recognition evaluation

To ensure human-aligned fine-tuning does not lead to catastrophic forgetting on the original task, we evaluate the baseline and fine-tuned video models’ action recognition performance, following the UCF101 benchmark (Soomro et al., 2012) split1 (101 action categories). We freeze the model backbones (both pretrained and fine-tuned with LORA adapters), extract model embeddings, and train a linear probe on UCF101 split1 across three seeds (Top-1 accuracy; mean±sd, see Appendix D).

## 4 Results

### Q1: Do Pretrained Models Capture Human-Perceived Similarity?

**Figure 2:**
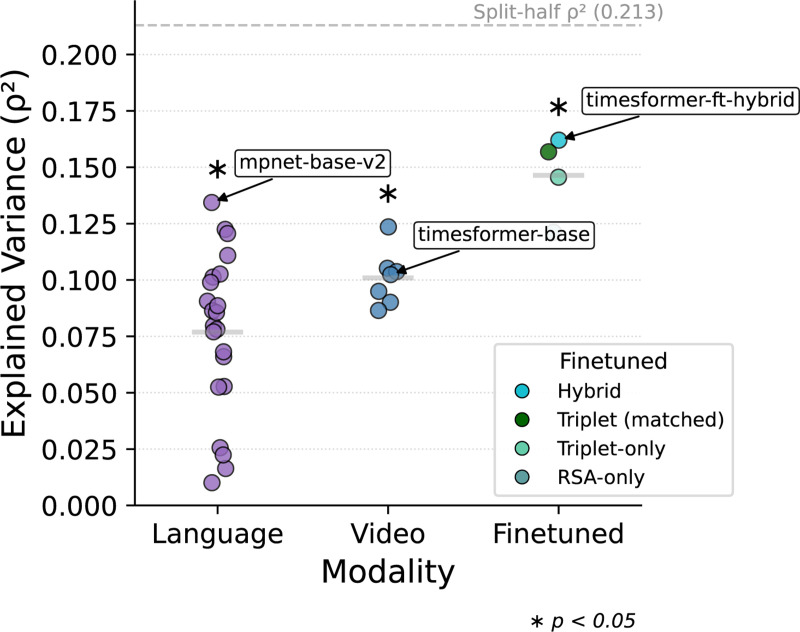
Explained variance $\left(R^{2}\right)$ computed as Spearman’s rank correlation between model embeddings and human similarity judgments and we report its square as a measure of explained variance (differing from regression). Language models outperform pretrained video models, but fine-tuned TimeSformer exceeds both. Horizontal dashed line shows the split-half spearman correlation^2^ of the human RSM used as our noise ceiling (Appendix §B.3).

On average, both language and video models show a modest ability to capture human video similarity judgments (Fig. 2). Among pretrained baselines, the best caption–based language embedding (*paraphrase-multilingualmpnet-base-v2*) achieves higher explained variance $\left(R^{2}=0.134\right)$ *and* higher OOO accuracy (70.38%) than the best pretrained video model (TimeSformer: $R^{2}=0.102$; OOO = 63.59%; Appendix Tab. 2). Thus, even though human participants performed a purely visual task without captions, their judgments were better predicted by text embeddings, suggesting critical gaps in pretrained video models.

### Q2: How Can Video Models Learn Human-Like Similarity?

We next ask whether we can imbue video models with more human-like similarity structure via fine-tuning. To use the LORA procedure (which relies on a transformer architecture, see §3: Methods), we select TimeSformer as the best performing transformer model. Fine-tuning with hybrid triplet-RSA loss shows a significant improvement over the pretrained TimeSformer baseline in terms of both correlation and accuracy. Importantly, the hybrid fine-tuned video model outperforms all pre-trained models, including the best *language-based caption embeddings* both in terms of $R^{2}$ and OOO accuracy (Fig. 2; Appendix Tab. 2).

The hybrid loss also outperforms both the triplet-only and RSA-only fine-tuning, showing that the combination of local and global constraints is more effective than either alone (Fig. 2). Importantly, the triplet-budget-matched control achieved performance better than triplet-only but below hybrid, demonstrating that RSA contributes more than simply additional training signal.

In the **pretrained (baseline) case** (left), the video model contributes little unique variance, with most of its explanatory power overlapping with the language model and the language model still accounting for substantial unique variance on its own. In the **fine-tuned case** (right), shared variance between models increases and the video model captures more unique variance (see Appendix Tab. 3). These results suggest that fine-tuning both aligns the video model more closely with language-derived semantic structure and enables it to encode additional social–visual nuances that are less easily captured by caption embeddings.

#### Encoding of Social-Affective Attributes.

To test whether fine-tuning enhances the encoding of social and emotional factors of the videos, we run linear probes predicting five attributes often emphasized in human descriptions of social interaction: intimacy, valence, arousal, dominance, and communication.

**Figure 4:**
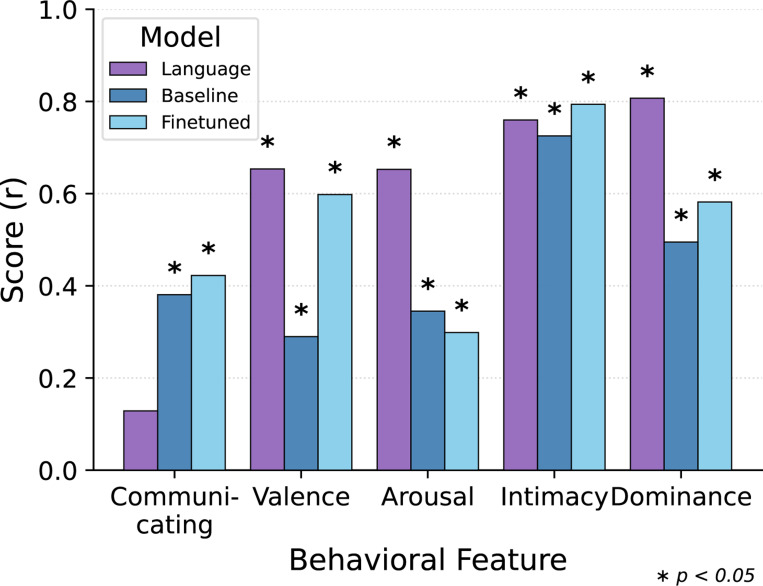
Pearson correlation (r) scores for predicting social-affective attributes from video embeddings using Ridge Regression. Language (purple) is the best performing language model for comparison to baseline (dark blue) and finetuned (light blue) TimeSformer.

As shown in Fig. 4, fine-tuning substantially improves the model’s sensitivity to social-affective dimensions. The largest gains appear in *Valence* and *Dominance*, while *Intimacy* was already well-encoded even before fine-tuning. *Communicating* shows modest improvement, whereas *Arousal* remains relatively unchanged. Notably, the model was never trained on these human judgments. Its improvement therefore suggests that human similarity judgments were themselves shaped by these underlying factors, and highlights how similarity-based supervision encourages the emergence of interpretable, socially meaningful features.

#### Action-recognition performance

On UCF101, the pretrained TimeSformer achieved 95.75 ± 0.18% Top-1 accuracy with a frozen linear probe across three seeds, and the fine-tuned model achieved 95.70 ± 0.14%. The negligible difference (paired mean Δ=−0.05pp) confirms that behavior-guided fine-tuning preserves action recognition ability, with no catastrophic forgetting.

## 5 Discussion

Our findings reveal a substantial mismatch between how current video models and humans perceive social video clips, and demonstrate a practical route to reduce this gap via behavior-guided fine-tuning. We created a new dataset of human video similarity judgments and presented an approach to align video model representations with humans. We found that while pretrained video models already capture some aspects of human similarity, they lag behind language-based embeddings. To close this gap, we fine-tuned a video transformer using a combination of triplet and RSA losses derived from human judgments, resulting in a model that more closely reflects human notions of similarity. This fine-tuned model not only aligns better with human judgments in aggregate, but also generalizes to better match judgments of high-level social-affective concepts, as evidenced by linear probe analyses. Variance partitioning further revealed that fine-tuning shifted the video model toward the semantic structure captured by language model embeddings while also contributing unique explanatory variance not captured by language models, indicating a unique contribution of visual information to this task.

### 5.1 Human Alignment as Supervision

Our approach frames human similarity judgments as a distinct form of supervision: instead of predicting explicit labels, the model is guided to organize its representation space to mirror human relational structure. This complements categorical labels by encouraging the geometry to capture factors humans intuitively use, such as social or affective context. Compared to alternatives like attribute annotation (e.g., intimacy or scenario type), this method is holistic: humans integrate multiple cues when judging similarity, and alignment recovers that integrated structure without enumerating each factor. Our social probe experiments also showed the fine-tuned model learned attributes it was never directly trained on. Interestingly, prior work has shown that video models struggle to match these attributes (Garcia et al., 2025), highlighting a particular benefit of fine-tuning for improving social judgments. Similar benefits from human similarity supervision have been demonstrated in prior work (Muttenthaler et al., 2023; Fu et al., 2023); our study extends these findings to social videos, areas that AI vision typically struggles with (Garcia et al., 2025).

### 5.2 Why Language Models Outperformed Video Models

Understanding social interactions often requires abstract inferences (goals, roles, affect) that go beyond visible motion. Video models, trained mainly for action classification, may emphasize kinematics and object cues, while caption-based language embeddings encode high-level semantics (e.g., “friends boxing for fun” vs. “strangers fighting angrily”). Humans likely rely on similar latent variables, which explains why language embeddings aligned more closely with human judgments. However, the fact that these are learnable by a video model, and that a fine-tuned video model can learn to explain human variance not attributable to language models, supports the idea that humans encode many aspects of this social structure visually (McMahon & Isik, 2023). An open question is whether self-supervised video models trained via predictive representation learning may close this gap: recent work such as V-JEPA 2 (Assran et al., 2025) suggests promising progress in this direction. Comparing more modern video models to this dynamic human benchmark is a promising area for future video model evaluation.

#### On the Hybrid Loss.

Our fine-tuning objective combines a triplet loss with an RSA loss, balancing local and global alignment. The triplet component ensures that fine-grained distinctions from the original model are preserved while pulling together pairs judged similar by humans. The RSA component complements this by aligning the model’s overall pairwise structure with human RSMs, distilling relational knowledge at a global level. This echoes findings by Muttenthaler et al. (2023), who showed that constraining global geometry to match human similarity can yield more interpretable and task-effective features when local structure is preserved. Our contribution goes further by introducing RSA as a training signal: whereas RSA is usually used as an analysis tool (Kriegeskorte et al., 2008), we re-purpose it as a differentiable objective. Together, the hybrid loss leverages local and global supervision to nudge the representation toward the richer semantic space reflected in human judgments.

### 5.3 Limitations

#### Dataset coverage.

The 250 videos in our dataset, though diverse, originate from a single source corpus. Stronger robustness claims require testing transfer to other video datasets and domains, especially those with different styles, contexts, and cultural settings. Evaluating cross-dataset generalization will be important for assessing the broader applicability of human-aligned representations. The high prediction accuracy of our fine-tuned model suggests it may be used as a tool to generate synthetic similarity data on larger scale video datasets.

#### Evaluator subjectivity.

Social similarity judgments inherently vary across individuals due to differences in cultural background, personal experience, and attentional focus. Our current model captures only the aggregate consensus, which smooths over such heterogeneity. While this is useful for deriving a stable group-level metric, it limits personalization. Future work could explore individualized alignment by collecting repeated judgments from single users or by clustering annotators with similar perceptual styles, enabling models that reflect user-specific or subgroup-specific social perception.

#### Task scope.

We primarily evaluate similarity alignment and a few attribute probes. Although preliminary checks suggest that the fine-tuned model remains competent on basic action recognition, it leaves open the possibility of trade-offs: enhancing human alignment could in principle reduce discriminative power on conventional benchmarks. Addressing this will require more comprehensive evaluations across multiple tasks and domains. Multi-objective training (i.e., combining classification loss with alignment losses) offers a principled safeguard, ensuring that models retain conventional task performance while gaining alignment with human similarity structure.

### 5.4 Broader Impact

Aligning video models with human social similarity judgments offers a pathway to more intuitive and trustworthy AI systems. Human-aligned embeddings could improve video retrieval, recommendation, and interpretability by organizing content in ways that reflect human categorization. Our findings suggest that such alignment also promotes emergent encoding of social-affective features, with potential applications in affective computing and safety-sensitive domains. However, models that reflect human perception may also inherit human biases. Our dataset—while diverse—may encode culturally specific notions of similarity. Broader deployments should include bias analysis and diverse annotation sources to ensure fairness and robustness across populations.

## 6 Conclusion

We integrate ideas from cognitive science and deep learning to enforce a human-aligned representational structure that was previously absent in video models. The success of this approach in the social video domain suggests broader applicability. As AI systems interact with human preferences and categorization (whether in recommending media, assisting decision-making, or understanding user behavior), having their internal representations align with how humans naturally structure the world will be invaluable. We hope this inspires future work to further explore human-aligned model training—bringing machine representations a step closer to human mental representations, and thereby making AI systems more interpretable and effective in human-centric tasks.

## Supplementary Material

Supplement 1

## Figures and Tables

**Figure 3:**
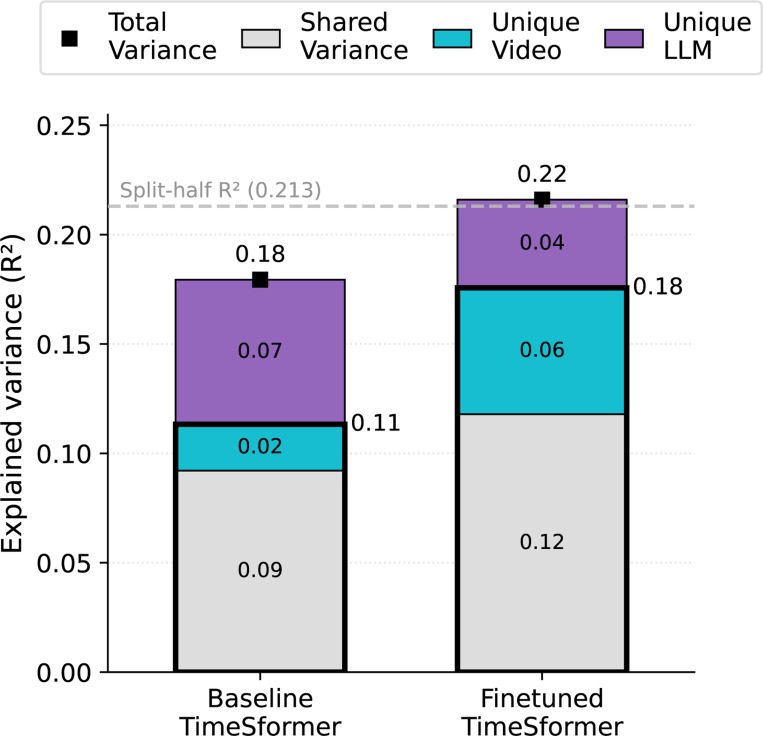
Variance partitioning before and after fine-tuning. Fine-tuning increases the unique variance explained by the TimeSformer (cyan), reduces the unique contribution of the language model (purple), and expands shared variance (gray). This shows that the fine-tuned video model both captures variance previously available only from captions and better overlaps with language-based structure. Total variance explained (black markers) approaches the reliability ceiling. Thicker black outline shows total variance explained by the video model.

## Reproducibility Statement

We emphasize transparency and full reproducibility of all results. We will release the video captions and ∼49k human odd-one-out judgments with the official 200/50 split, along with human RSMs and annotator counts. All evaluated models, preprocessing steps, and metrics are documented in § 3, with RSA and variance partitioning analyses detailed in Appendix B.2; code will be provided for embedding extraction, similarity computation, and evaluation of $R^{2}$ and OOO accuracy (see Appendix C). Training details for TimeSformer with LORA adapters are described in § 3, and we will release configuration files and scripts for the hybrid, triplet-only, RSA-only, and triplet-budget-matched models. Validation protocol and reporting metrics follow the procedure in § 3.3.3-§ 4. The UCF101 linear-probe action recognition evaluation and social-affective probing experiments are documented in Section D, with full pre-processing and training scripts to be released. Finally, all training and evaluation code, pretrained adapters, and precomputed RSMs will be made available to support both full retraining and lightweight reproduction.
