# Supplementary material for: Aligning Video Models with Human Social Judgments via Behavior-Guided Fine-Tuning
Source: ArXiv. 2025 Oct 1:arXiv:2510.01502v1. Preprint. [Version 1] (PMC12622209)
Supplement: Supplement 1 [file NIHPP2510.01502v1-supplement-1.pdf]

A Triplet Selection Algorithm

Because the triplet sample is sparse relative to all $\binom{250}{2}$ pairs, $\mathbf{S}^{(human)}$ is an aggregate estimate rather than a fully observed matrix. To ensure adequate coverage, we designed the triplet selection procedure so that *every possible pair of videos appears in at least one triplet*. This guarantees that each pair receives at least one human rating, providing a principled basis for constructing the similarity matrix while keeping participant requirements manageable.

Conceptually, this problem is equivalent to a *set cover*: the universe of elements consists of all pairs of videos, and each triplet corresponds to a subset that covers three of those pairs. Finding the truly minimal set of triplets that covers all pairs is NP-hard. Instead, we employed a **greedy approximation strategy**, which iteratively chooses the most informative triplet at each step:

- At each iteration, we randomly sample a candidate pool of triplets.
- From this pool, we select the triplet that covers the largest number of pairs not yet included.
- We then mark those pairs as covered and continue until every pair has been assigned to at least one triplet.

This greedy search prioritizes efficiency: it minimizes the number of triplets (and thus participant ratings) required to guarantee full pairwise coverage. After coverage is achieved, we adjust the total number of triplets so that it is divisible by 220, corresponding to a balanced design in which each participant contributes 22 trials.

Algorithm 1 Triplet Selection Covering All Pairs (Greedy Set Cover Approximation)

Require: Number of items N (e.g., $N = 250$ for 250 video stimuli)
Ensure: Set of triplets T covering all pairs, with $|T|$ divisible by 220

$P \leftarrow \{(i, j) \mid 0 \leq i < j < N\}$ ▷ All pairs
$S \leftarrow \{(i, j, k) \mid 0 \leq i < j < k < N\}$ ▷ All triplets
$T \leftarrow \emptyset$ ▷ Selected triplets
while $P \neq \emptyset$ **do**
    $C \leftarrow$ random sample of $\min(|S|, 10,000)$ triplets from S
    $best\_triplet \leftarrow$ triplet in C maximizing coverage w.r.t. P
    $T \leftarrow T \cup \{best\_triplet\}$
    Remove all pairs in $best\_triplet$ from P
end while
$r \leftarrow |T| \bmod 220$
if $r \neq 0$ **then**
    Sample $220 - r$ triplets randomly from S and add to T
end if
return T

B Supplementary Evaluation and Analysis Procedures

B.1 RSA objective

During training we use Pearson-correlation RSA on z-scored pairwise distances. Pearson is smooth, so gradients propagate from the correlation through distances back to the embeddings. (For evaluation we report Spearman ρ^2, which is rank-based and non-differentiable.)

B.2 Variance partitioning analysis

We model human distances $d_{\text{human}}(i, j)$ with multiple regression using model distances as predictors. For models $X_1, X_2, \ldots$, we fit

$$\hat{d}(i,j) \;=\; \beta_0 \;+\; \sum_m \beta_m \, d_{X_m}(i,j)$$

over all video pairs in the test split, and report R^2. Unique and shared contributions are obtained by comparing nested models (e.g., unique X_1 is $R^2_{X_1,X_2} - R^2_{X_2}$); confidence intervals are computed via bootstrap over pairs. We use the best language model as one predictor, and the pretrained and fine-tuned TimeSformer as the other predictors.

B.3 Split–half reliability

We estimate a noise ceiling for the human RSM with a split–half procedure that respects unequal judgments per pair. In each of 1,000 iterations we: (1) restrict to lower-triangle pairs with at least two ratings; (2) reconstruct binary votes ("similar"/"dissimilar") for each pair using its observed proportion and count, shuffle, and split the votes into two halves; (3) compute the proportion "similar" in each half for every pair and take the Spearman correlation across pairs between halves; (4) average these correlations over iterations and apply the Spearman–Brown correction to estimate full-sample reliability. We report this corrected average as the split–half noise ceiling for the human judgments. In figures, we label this as *split–half* R^2, i.e., the squared Spearman–Brown–corrected split–half correlation.

C Code and data availability

All code used in this paper and our sentence captions are publicly available: (`https://github.com/garciakathy/similarity-judgments-finetuning`). The videos shown to participants for the triplet OOO similarity judgments task and therefore are from the Moments in Time (MiT) dataset (`http://moments.csail.mit.edu`). The MiT license restricts public release of videos from the dataset, and so we ask to please contact the authors for access.

D Action Recognition Performance

We include here the full results of the UCF101 linear-probe evaluation. All backbone parameters were frozen, and a linear classifier was trained on top of `[CLS]` features extracted from the pretrained and fine-tuned TimeSformer models. Training was repeated across three random seeds, and Top-1 accuracy is reported as mean $\pm$ standard deviation.

Table 1: Linear probe Top-1 accuracy (%) on UCF101 split1 with frozen backbones. Reported as mean $\pm$ standard deviation over 3 seeds.

Backbone	Top-1 (%)
Pretrained	95.75 ± 0.18
Fine-tuned	95.70 ± 0.14

E Model Performance and Supervision Budget

Matching Constraints. Despite the same number of optimizer steps across all approaches, the hybrid objective includes an additional RSA term, introducing a modest number of extra supervision signals ($\approx$ 738 pairwise constraints per epoch) beyond the triplet loss (12,240 pairwise constraints). To ensure a fair comparison, we trained a *triplet-only (budget-matched)* variant by adding the same number of extra triplet constraints each epoch. This budget-matched triplet model slightly outperforms standard triplet-only training, confirming that more constraints help. Yet, it still underperforms compared to the hybrid model, indicating that the RSA term contributes qualitatively different information by enforcing global structure beyond what can be achieved by simply adding more triplet comparisons.

Table 2: Model performance and supervision constraints budget (— indicates not applicable).

Model UID	Explained Variance (R^2)	OOO Accuracy	Constraints/epoch
Finetuned/Base TimeSformer Models			
timesformer-ft-hybrid	0.162023	74.46%	12978
timesformer-ft-triplet-match	0.156857	66.58%	12978
timesformer-ft-triplet	0.145600	70.65%	12240
timesformer-ft-rsa	0.121153	63.86%	13038
timesformer-base	0.102408	63.59%	—
Video Models			
x3d-m	0.123559	68.48%	—
x3d-s	0.105202	64.67%	—
x3d-xs	0.103721	64.95%	—
i3d-r50	0.094969	67.66%	—
c2d-r50	0.090121	65.76%	—
slow-r50	0.086501	67.93%	—
slowfast-r50	0.085466	64.95%	—
Language Models			
paraphrase-multilingual-mpnet-base-v2	0.134374	70.38%	—
mxbai-embed-2d-large-v1	0.122445	66.58%	—
paraphrase-multilingual-MiniLM-L12-v2	0.120615	67.39%	—
distiluse-base-multilingual-cased-v1	0.110899	64.95%	—
paraphrase-MiniLM-L6-v2	0.102647	65.49%	—
all-distilroberta-v1	0.101303	63.04%	—
stsb-distilroberta-base-v2	0.098953	64.13%	—
mxbai-embed-large-v1	0.090592	67.39%	—
all-roberta-large-v1	0.088598	63.04%	—
all-mpnet-base-v1	0.086371	66.58%	—
all-mpnet-base-v2	0.085562	64.67%	—
all-MiniLM-L6-v1	0.078124	65.22%	—
all-MiniLM-L6-v2	0.077037	65.49%	—
multi-qa-MiniLM-L6-cos-v1	0.068142	64.40%	—
all-MiniLM-L12-v2	0.065997	67.39%	—
LaBSE	0.052770	61.96%	—
clip-ViT-B-32-multilingual-v1	0.052506	62.77%	—
FacebookAI/roberta-base	0.025612	59.24%	—
FacebookAI/xlm-roberta-base	0.022418	49.46%	—
FacebookAI/roberta-large-mnli	0.016395	47.83%	—
FacebookAI/xlm-roberta-large	0.010090	57.07%	—

Table 3: Subset: Finetuned TimeSformer along with best Video and Language model performance.

Model UID	Explained Variance (R^2)	OOO Accuracy
Finetuned/Base TimeSformer		
timesformer-ft-hybrid	0.162023	74.46%
timesformer-ft-triplet-match	0.156857	66.58%
timesformer-ft-triplet	0.145600	70.65%
timesformer-ft-rsa	0.121153	63.86%
timesformer-base	0.102408	63.59%
Best Video Model		
x3d-m	0.123559	68.48%
Best Language Model		
paraphrase-multilingual-mpnet-base-v2	0.134374	70.38%
